# Comparison of the effects of kilohertz- and low-frequency electric stimulations: A systematic review with meta-analysis

**DOI:** 10.1371/journal.pone.0195236

**Published:** 2018-04-24

**Authors:** Hirotaka Iijima, Masaki Takahashi, Yuto Tashiro, Tomoki Aoyama

**Affiliations:** 1 Department of System Design Engineering, Keio University, Yokohama, Japan; 2 Department of Physical Therapy, Human Health Sciences, Graduate School of Medicine, Kyoto University, Kyoto, Japan; 3 Japan Society for the Promotion of Science, Tokyo, Japan; Toronto Rehabilitation Institute - UHN, CANADA

## Abstract

**Objective:**

This study aimed to determine whether kilohertz-frequency alternating current (KFAC) is superior to low-frequency pulsed current (PC) in increasing muscle-evoked torque and lessening discomfort.

**Data sources:**

The electronic databases PubMed, PEDro, CINAHL, and CENTRAL were searched for related articles, published before August 2017. Furthermore, citation search was performed on the original record using Web of Science.

**Review methods:**

Randomized controlled trials, quasi-experimental studies, and within-subject repeated studies evaluating and comparing KFAC and PC treatments were included. The pooled standardized mean differences (SMDs) of KFAC and PC treatments, with 95% confidence intervals (CIs), were calculated using the random effects model.

**Results:**

In total, 1148 potentially relevant articles were selected, of which 14 articles with within-subject repeated designs (271 participants, mean age: 26.4 years) met the inclusion criteria. KFAC did not significantly increase muscle-evoked torque, compared to PC (pooled SMD: -0.25; 95% CI: -0.53, 0.06; *P* = 0.120). KFAC had comparable discomfort compared to that experienced using PC (pooled SMD: -0.06; 95% CI: -0.50, 0.38; *P* = 0.800). These estimates of the effects had a high risk of bias, as assessed using the Downs and Black scale, and were highly heterogeneous studies.

**Conclusions:**

This meta-analysis does not establish that KFAC is superior to PC in increasing muscle-evoked torque and lessening discomfort level. However, no strong conclusion could be drawn because of a high risk of bias and a large amount of heterogeneity. High quality studies comparing the efficacy between PC and KFAC treatments with consideration of potential confounders is warranted to facilitate the development of effective treatment.

## 1. Introduction

Neuromuscular electrical stimulation (NMES) is used in the clinical setting of rehabilitation, primarily to enhance muscle strength, particularly in subjects who are unable to perform conventional exercise.[1] NMES can be used to produce a muscle contraction equivalent to 40–60% of maximum voluntary isometric contraction (%MVIC).[2] The level of %MVIC is reportedly associated with the cross-sectional area of the activated skeletal muscle,[3] and current intensity is linearly related to NMES-evoked muscle torque.[2] Thus, NMES current intensity should be as high as possible to stimulate a broader cross-sectional area of the muscle and to produce muscle-evoked torque. However, the strong discomfort associated with NMES limits the dosage and consequently impedes the attainment of optimal muscle contraction,[4] highlighting the importance of suppressing the discomfort during NMES during rehabilitation.

The two major frequently used NMES approaches are low-frequency pulsed current (PC) and kilohertz-frequency alternating current (KFAC), commonly referred to as Russian current (i.e., frequency of 2500 Hz applied in 50 Hz rectangular bursts/s with a burst duty cycle of 50%).[5] PC and KFAC are delivered in the 1–100 Hz and 1–10 kHz frequency ranges, respectively. The most commonly used KFAC frequencies are 2.5 and 4 kHz.[6] Theoretically, KFAC has the advantage of lower skin impedance,[7] which allows more electrical energy to stimulate the skeletal muscle and generates stronger muscle contractions with less discomfort. A recent meta-analysis from seven studies with 127 subjects investigated which NMES protocol is better at producing quadriceps muscle-evoked torque and lessening the discomfort level related to NMES.[8] KFAC and PC had similar effects in producing muscle torque and discomfort level in healthy individuals.[8] This meta-analysis raised several experimental biases in the included studies, which would accelerate additional research in the rehabilitation field as reported by a recent article.[9] However, this meta-analysis has several methodological issues that need to be resolved. For example, a large heterogeneity existed among included subjects, however approaches to explore the cause of heterogeneity, such as subgroup and meta-regression analyses, were not performed. These points are important, because confidence in the effects estimate from meta-analysis depends on the quality of the included studies and analytic process of the meta-analysis,[10] as the former can be evaluated by the Grades of Recommendation, Assessment, Development, and Evaluation (GRADE) approach.[11] It is also important to emphasize that the effects estimate comparing KFAC and PC treatments in the previous meta-analysis had wide 95% confidence intervals (CIs),[8] which could change the clinical decision if the true effects estimates are in the upper or lower boundary.[12] Recently, additional relevant articles were reported [13, 14]; therefore, precision of effects estimate would be increased by synthesizing all these articles. These updated information would be helpful for clinicians and physical therapists in making and improving evidence-based treatment regimen using NMES for muscle strength.

Thus, the purpose of this systematic review was to update the meta-analysis examining current evidence that compared the effects of KFAC and PC on muscle-evoked torque and discomfort in adults, using the GRADE approach. We hypothesized that KFAC is superior to PC in increasing muscle-evoked torque and lessening discomfort level.

## 2. Materials and methods

This study was conducted according to the Preferred Reporting Items for Systematic Reviews and Meta-Analyses (PRISMA) statement,[15] PRISMA protocols (PRISMA-P),[16] Meta-analysis of Observational Studies in Epidemiology (MOOSE) checklist,[17] and Cochrane Handbook for Systematic Reviews of Interventions (S1 and S2 Files).[18] A detailed protocol for this systematic review has not been previously published and registered.

### 2. 1 Literature search and study selection

The electronic databases of PubMed, Physiotherapy Evidence Database (PEDro), Cumulative Index to Nursing and Allied Health Literature (CINAHL), and Cochrane Central Register of Controlled Trials (CENTRAL) were utilized. Searches used combined key terms, including “electric stimulation” and “adverse effects,” which use Medical Subject Headings terms. A database search strategy is provided in Method A in S3 File. These keywords include those used in the previous meta-analysis.[8] Google Scholar was also used as a complementary search engine. In addition, a manual search of the reference lists of past systematic reviews was performed. Furthermore, citation searching was performed on the original record using the Web of Science. These citation indices are recommended by the Cochrane Handbook.[18]

Studies that were included were (i) published in a peer review journal, (ii) written in English, (iii) had a randomized controlled trial (RCT), or quasi-experimental, and within-subject repeated design, (iv) had a treatment strategy that included KFAC and PC stimulations targeting the skeletal muscles, and (v) whose outcome included muscle performance and/or discomfort level. No restrictions were imposed on study dates, follow-up duration, target muscle, and subject characteristics. Although a previous meta-analysis included only healthy adults,[8] we also included adults other than healthy individuals, because NMES is used in rehabilitation, primarily, to enhance muscle strength particularly in subjects who are unable or unwilling to perform conventional exercises.[1] This is important given the GRADE approach criteria (i.e., indirectness).[19] For each electronic database, the endpoint was August 2017. Studies that investigated KFAC using interferential current were excluded because of its different mechanism from that of Russian currents,[6] as done in the previous meta-analysis.[8]

### 2. 2 Determining inclusion

One reviewer, who was also a content expert, assessed eligibility in accordance with the Cochrane Handbook.[18] The reviewer screened the title and abstracts yielded by the search. Full manuscripts of the articles that met the eligibility criteria were then obtained and reviewed. During these processes, the reviewer prepared and used simple predesigned Google spreadsheets to assess eligibility by extracting study features.

### 2. 3 Outcome measures and data extraction

The primary outcomes in this review were (i) skeletal muscle-evoked torque evaluated using %MVIC and (ii) discomfort level due to the electric stimulation treatment evaluated using a visual analog scale (VAS). When %MVIC was not available, other outcome measures of muscle performance, such as muscle-evoked torque that is non-normalized by MVIC, were used. The same reviewer collected the data using standardized data extraction form regarding authors, years, study design, subject population, electric stimulation parameters (i.e., frequency, pulse duration, current intensity), target muscle, outcome, and funding sources.

### 2. 4 Risk of bias assessment of included studies

The same reviewer evaluated the risk of bias of each study using the Downs and Black scale[20] that was slightly modified to include only 13 variables (bias: 7 items; confounding: 6 items) to assess internal validity (minimum: 0 point; maximum: 13 points) as in other meta-analyses.[21] This scale is a useful tool for assessing risk of bias in observational studies[22] and the methodological quality of both RCT and non-RCT of treatment.[18] This scale has been ranked in the top six quality assessment scales suitable for the use in systematic reviews for non-RCT.[22, 23] All items were scored 1 for fulfilling the criterion or 0 if the criterion was not filled. Publication that did not provide sufficient details to fulfill the criterion were also given a 0 score for being unable to be determined in accordance of the original index of Downs and Black scale. To assess intra-rater reliability in the current data set, the same examiner rescored more than 1 week after the first assessment. The intra-rater reliability was excellent[24] for all 13 items (*κ* = 0.967, 95% CI: 0.929–1.000), for bias-related 7 items (*κ* = 0.976, 95% CI: 0.930–1.000), and confounding-related 6 items (*κ* = 0.919, 95% CI: 0.808–1.000).

### 2. 5 Data analysis

For the meta-analysis, pooled estimates and 95% CIs for standardized mean differences (SMD) were calculated using the DerSimonian-Laird method[25] (Method B in S3 File). The SMD was calculated for paired samples using the mean difference between-group (PC and KFAC) divided by the pooled standard deviation (SD). The formulae for calculating the pooled SD and pooled SMD are shown in eMethods in the Supplement. The meta-analyses were performed using Review Manager (RevMan) Version 5.3 (the Nordic Cochrane Centre, the Cochrane Collaboration, Copenhagen, Denmark). A forest plot was used to represent the results of the meta-analysis in accordance with a previous study.[26] The size of the SMD was interpreted using Cohen’s d[27] (<0.5: small effect size, 0.5–0.8: moderate effect size, and ≥0.8: large effect size). When mean and SD values were not directly reported in an article, they were calculated from other available data, if possible; for example, mean and SD values were estimated from the figure in each article. For the significant findings reported with an exact *P*-value (i.e., *P* = 0.037, not *P* < 0.05), SD was calculated using RevMan calculator. The calculator can provide standard deviations from available data even if the actual standard deviations are missed in each included article. For studies with multiple evaluation points, data from the last available evaluation point was used, as in previous meta-analyses.[21] To evaluate the robustness of this imputation method, prespecified sensitivity analysis was performed while excluding studies with imputed data. The last evaluation point would be variable because it would represent the point at which the greatest difference of muscle performance and discomfort level between KFAC and PC is observed. To provide SMD with 95% CI in specific subjects and skeletal muscles, certain post hoc sensitivity analyses were also performed: (i) excluding articles that included subjects other than healthy adults and (ii) excluding articles that targeted muscles other than the quadriceps muscle.

To test for publication bias (funnel plot asymmetry), a funnel plot and Egger’s test[28] (Method C in S3 File) were used. A test for funnel plot asymmetry formally examines whether the association between estimated intervention effects and a measure of study size (standard error of the intervention effect) is greater than might be expected to occur by chance. *P* < 0.10 indicated the existence of publication bias, as practiced by a previous study.[28]

Study heterogeneity was assessed using *I*^*2*^ and Q statistics.[29] If *I*^*2*^ was ≥50%, random effects meta-regression was performed using certain parameters selected *a priori*: (i) age (per year), (ii) %female (per percent), (iii) BMI (per unit), (iv) year of publication (per year), (v) Downs and Black scale score (per point), and (vi) funding source (0: no, 1: yes). These factors were chosen because of their potential association with the effects estimate of NMES and primary outcomes and not on the causal pathway between NMES and each outcome.[30, 31] All other statistical analyses were performed using JMP Pro 12.2 (SAS Institute, 100 SAS Campus Drive Cary, NC 27513–2414, USA). *P* < 0.05 was considered statistically significant.

### 2. 6 Quality assessment of body of evidence: GRADE approach

One reviewer graded the quality of the outcome measures of interest in accordance with the GRADE approach[11] as high, moderate, low, and very low using the following five domains: risk of bias, inconsistency, indirectness, imprecision, and publication bias. The evidence quality was downgraded if (i) primary outcomes have a high risk of bias; we defined this as Downs and Black scale score of <8 points because most of the trials in the meta-analysis have a conservative approach (i.e., severe judgment to prevent overestimation); (ii) heterogeneity between trials was more than substantial (*I*^*2*^ ≥50%)[18, 32]; (iii) included subjects in most of the trials were healthy adults (i.e., indirectness in the population)[19], (iv) the 95% CI of SMD was large; that is, clinical action differs if the 95% CI is in the upper or lower boundary [12]; and (v) publication bias, funnel plot asymmetry, existed as evaluated by the Egger’s regression test[33].

## 3. Results

### 3. 1 Study selection

**Fig 1:**
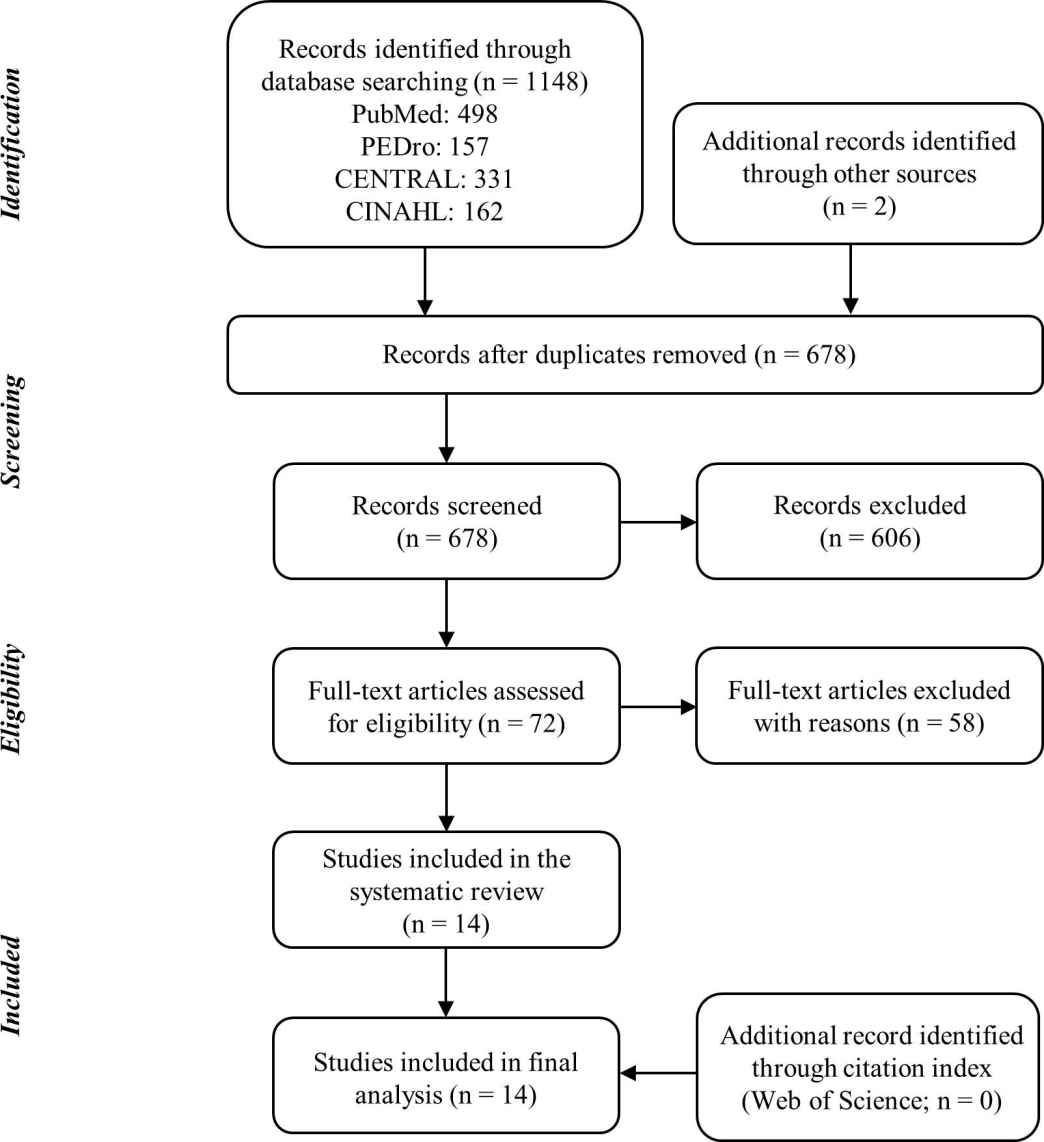
Review flow diagram.

The database search yielded 1148 studies. Fig 1 shows a flow chart of the study selection. After adjusting for duplicated studies, the titles and abstracts of 678 studies were screened, and the remaining 72 studies were assessed for eligibility by full-text screening. Finally, 14 studies met the eligibility criteria. Exclusion reasons for the 58 studies during the full-text screening were publication in a non-English language (n = 20; 35.7%), study design other than RCT, quasi-experimental design, or within-subject repeated designs (n = 15; 26.3%), treatment strategy other than KFAC and PC treatments (n = 22; 38.6%), and outcome measure other than muscle performance (n = 1; 1.8%). Percent agreement of full-text screening between the first and second assessments was 100.0%. The citation index found no additional articles; in total, 14 studies were used in the meta-analysis.

### 3. 2 Study characteristics

Table 1 shows the characteristics of the included studies that compared the effects of PC and KFAC. All included studies had a within-subject repeated design[9, 13, 14, 34–44]. A total of 271 subjects (mean age: 26.4 years) were included from 14 studies. Of the 12 studies that reported sex (n = 228 subjects), 131 (57.5%) were female. Of the studies included, 13 (92.9%) evaluated healthy adults without any musculoskeletal disease,[9, 13, 14, 34–41, 43, 44] and 1 study evaluated adults with spinal cord injury.[42] Of the 14 studies, 11 (78.6%) and 2 (14.3%) studies evaluated the quadriceps muscle and the wrist extensor muscle, respectively. Only 1 (7.1%) study evaluated the quadriceps, hamstrings, and gluteus muscles. Among the articles, 3 (21.4%) reported a funding source.[9, 42, 43].

**Table 1:** Summary of included studies.

Author	Subject Population	Frequency (Hz)	Pulse Duration (μs)	Intensity (mA)	Target Muscle	Outcome
***Within-subject repeated measure***						
Aldayel A, 2010[34]	Healthy adults (N = 12; age: 31.2 ± 5.5 y; weight: 81.4 ± 15.2 kg; height: 174.3 ± 4.8 cm; BMI: 26.9 kg/m^2^; 0% F)	PC: 75 KFAC: 2500	PC: 400 KFAC: 400	Maximum tolerance: PC: 84.4 ± 13.8 KFAC: 78.9 ± 4.0	Quadriceps	%MVIC
Aldayel A, 2011[35]	Healthy adults (N = 9; age: 34.0 ± 7.0 y; weight: 85.4 ± 14.1 kg; height: 174.0 ± 5.1 cm; BMI: 28.2 kg/m^2^; 0% F)	PC: 75 KFAC: 2500	PC: 400 KFAC: 400	Maximum tolerance: PC: 86.4 ± 13.6 KFAC: 80.0 ± 4.1	Quadriceps	%MVIC
Dantas LO, 2015[36]	Healthy adults (N = 23; age: 21.6 ± 2.5 y; weight: 58.8 ± 8.5 kg; height: 166.3 ± 7.3 cm; BMI: 21.3 kg/m^2^; 100% F)	PC1: 50 PC2: 50 KFAC1: 2500 KFAC2: 1000	PC1: 200 PC2: 500 KFAC1: 200 KFAC2: 500	Maximum tolerance	Quadriceps	%MVIC
Fukuda TY, 2013[13]	Healthy adults (N = 30; age: 25.0 ± 3.0 y; BMI: 24.2 ± 1.7 kg/m^2^; 0% F)	PC1: 50 PC2: 50 KFAC: 2500	PC1: 400 PC2: 400 KFAC: 400	PC1: 59.7 ± 10.9 PC2: 60.3 ± 15.0 KFAC: 74.7 ± 14.5	Quadriceps	%MVIC
Holcomb W, 2000[37]	Healthy adults (N = 10; age: 24.0 y; weight: 64.4 kg; height: 168.4 cm; BMI: 22.8 kg/m^2^; 50.0% F)	PC: 90 KFAC: 2500	PC: 200 ms KFAC: 5.6 ms (burst duration)	Maximum tolerance	Quadriceps	%MVIC
Laufer Y, 2001[44]	Healthy adults (male: N = 15; age: 30.7 ± 5.5 y; female: N = 15, age: 28.2 ± 5.2 y; 50% F)	PC: 50 KFAC: 2500	PC: 200 KFAC: 200	PC: 0–150 mA; KFAC: 0–100 mA	Quadriceps	%MVIC
Laufer Y, 2008[38]	Healthy adults (N = 26; age: 27.4 ± 5.0 y; BMI: 23.3 ± 3.3 kg/m^2^; 57.7% F)	PC: 50 KFAC: 2500	PC: 200 KFAC: 200	Maximum tolerance	Wrist extensor	%MVIC
Lein DH Jr, 2015[39]	Healthy adults (N = 12; age: 25.5 ± 9.0 y; weight: 74.4 ± 13.1 kg; height: 175.0 ± 10.4 cm; BMI: 24.3 kg/m^2^; 50.0% F)	PC: 10, 20, 30, 40, 50, 70 KFAC: 2500	PC: 200, 300, 400, 500 KFAC: 200	NA	Quadriceps	%MVIC
Medeiros FV, 2017[9]	Healthy adults (N = 25; age: 21.0 ± 3.0 y; weight: 59.0 ± 9.0 kg; height: 162.0 ± 5.0 cm; BMI: 22.5 kg/m^2^; 100% F)	PC1: 50 PC2: 50 KFAC1: 1000 KFAC2: 4000	PC1: 250 PC2: 500 KFAC1: 500 KFAC2: 250	Maximum tolerance: PC1: 110.0 ± 12.1 PC2: 72.4 ± 22.4 KFAC1: 108.8 ± 18.5 KFAC2: 82.6 ± 21.2	Quadriceps	%MVIC
Scott W, 2015[40]	Healthy adults (N = 12; age: 22.6 y; weight: 77.6 kg; height: 174.0 cm; BMI: 25.6 kg/m^2^; 100% F)	PC: 50 KFAC: 2500	PC: 500 KFAC: 200	NA	Quadriceps	%MVIC
Snyder-Mackler L, 1989[41]	Healthy adults (N = 12; age: 28.7 [21–40] y; 100% F)	PC: 50 KFAC: 2500	PC: 200 KFAC: 200	Maximum tolerance	Quadriceps	%MVIC
Szecsi J, 2007[42]	Spinal cord injury (N = 11; age: 35.5 ± 8.8 y; 27.3% F)	PC: 20 KFAC: 4000	PC: 500 KFAC: 10 ms (burst duration)	NA	Quadriceps, hamstrings, gluteus	Isometric torque
Vaz MA, 2012[14]	Healthy adults (N = 22; age: 25.0 ± 4.0; 59.1% F)	PC: 50 KFAC: 2500	PC: 400 KFAC: 10 ms (burst duration)	PC: 63.3 ± 10.0 KFAC: 73.3 ± 15.0	Quadriceps	%MVIC
Ward AR, 2006 [43]	Healthy adults (N = 32; age: 30.8 ± 14.5)	PC1: 50 PC2: 50 KFAC1: 2500 KFAC2: 2500	PC1: 200 PC2: 500 KFAC1: 200 KFAC2: 500	Maximum tolerance	Wrist extensor	%MVIC

BMI: body mass index; %F: % female; PC: pulsed current; KFAC: kilohertz frequency altering current; MVIC; maximum voluntary isometric contraction; NA: not applicable.

### 3. 3 NMES protocols

The most commonly used carrier frequency was 50 and 2500 Hz in PC (n = 10 [71.4%]) and KFAC (n = 12 [85.7%]), respectively. In KFAC, the carrier frequency was likely to be modulated at 50 Hz burst frequency (n = 11 [78.6%]). The pulse duration was variable and ranged 200–500 μs in both PC and KFAC. Only a few articles reported mean current intensity.[9, 13, 14, 34, 35] The mean current intensity was 59.7–110.0 mA and 73.3–108.8 mA for PC and KFAC, respectively.

### 3. 4 Risk of bias within studies

The included trials had a mean Downs and Black scale score of 5.9 ± 0.6 (range, 5–7) points, which indicates that the overall quality was poor (Table A in S3 File). None of the included trials blinded participants and assessors who measured key outcomes and concealed randomization of patients, and none had adequate adjustment for confounders.

### 3. 5 Outcome measures: PC vs. KFAC

#### 3. 5. 1 Muscle performance

**Fig 2:**
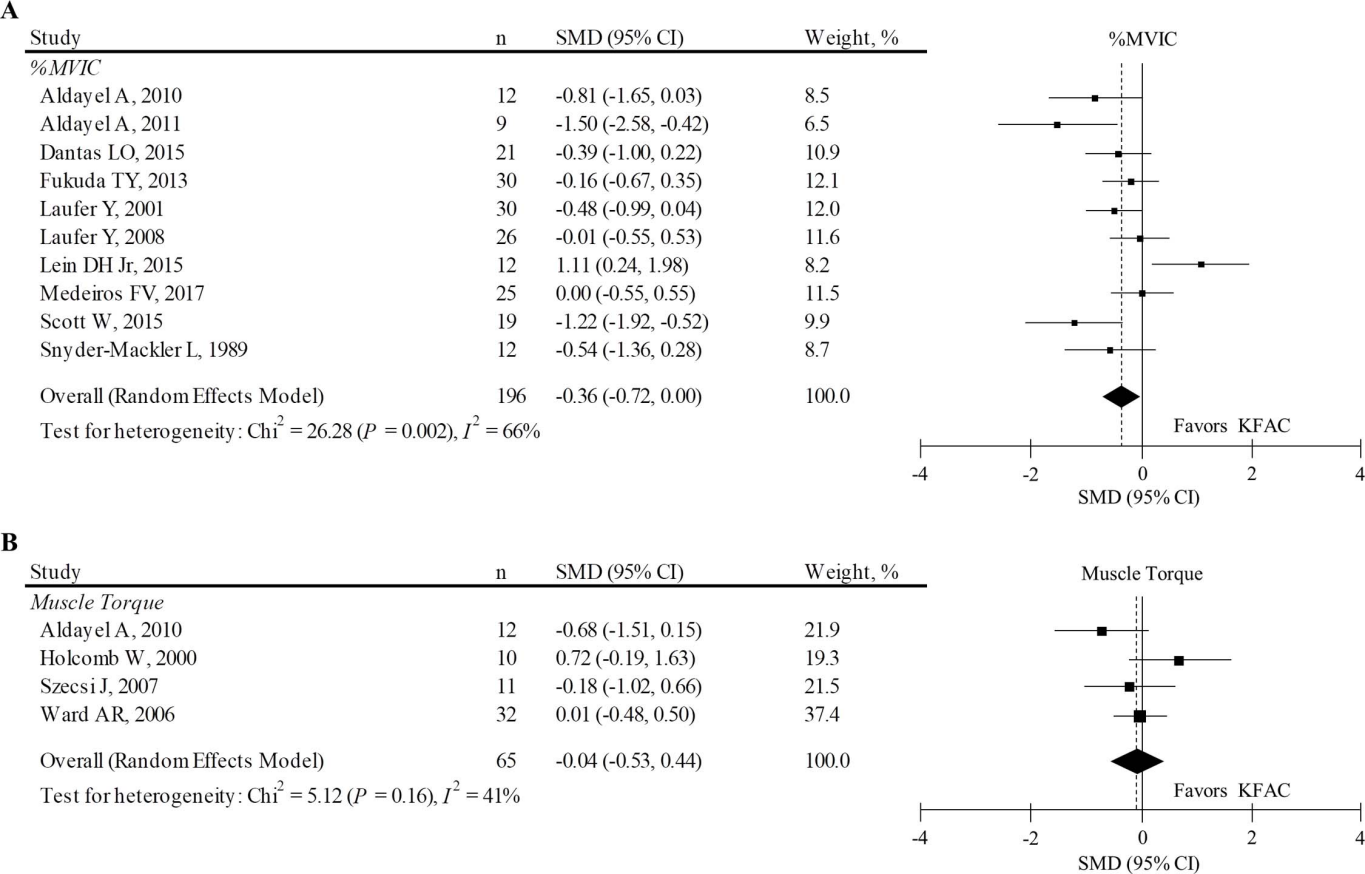
SMD and 95% CI for the muscle performance between PC and KFAC stimulations. **A.** %MVIC. **B.** Muscle evoked torque that is non-normalized data by MVIC. The diamond represents the pooled effect size using the DerSimonian-Laird method. The vertical solid line at 0 represents no difference. The vertical dotted line represents pooled SMD. SMD: standardized mean difference; CI: confidence interval; MVIC: maximum voluntary isometric contraction; PC: pulsed current; KFAC: kilohertz frequency alternating current.

A difference between PC and KFAC in the %MVIC (n = 196),[9, 13, 34–36, 38–41, 44] and muscle-evoked torque (n = 65)[34, 37, 42, 43] was observed in 10 and 4 articles, respectively. In 3 [9, 35, 39] and 2 [34, 43] studies, missing data were imputed from available data to estimate pooled SMD for %MVIC and muscle torque, respectively. Considering all studies, the pooled SMD on the muscle performance was calculated (Fig 2). The results reveal that KFAC led to a slightly lower %MVIC compared to PC (Fig 2A), although the difference was not statistically significant (pooled SMD: -0.36; 95% CI: -0.72, 0.00; *P* = 0.050). However, effects estimates were highly heterogeneous among studies (*I*^*2*^ = 66%; *P* = 0.002). Excluding 3 [9, 35, 39] studies with imputed data led to a significantly lower %MVIC in KFAC than those in PC (pooled SMD: -0.45; 95% CI: -0.75, -0.16; *P* = 0.002). Posthoc sensitivity analysis revealed that KFAC had significantly lower torque than PC when 1 article targeting the wrist extensor muscle[38] was excluded from the meta-analysis (pooled SMD: -0.41; 95% CI: -0.81, -0.01; *P* = 0.040).

On the other hand, KFAC led to a similar muscle torque compared to PC (Fig 2B). Excluding 2 [34, 43] studies with imputed data had a minimal effect on muscle torque. Posthoc sensitivity analysis showed comparable results even when 1 article, including subjects with spinal cord injury,[42] was excluded (data not shown). Another posthoc sensitivity analysis revealed that KFAC had significantly lower torque compared to PC when 2 articles, targeting the wrist extensor muscle[43] or hamstrings and gluteus in addition to the quadriceps muscles,[42] were excluded from the meta-analysis (data not shown).

**Fig 3:**
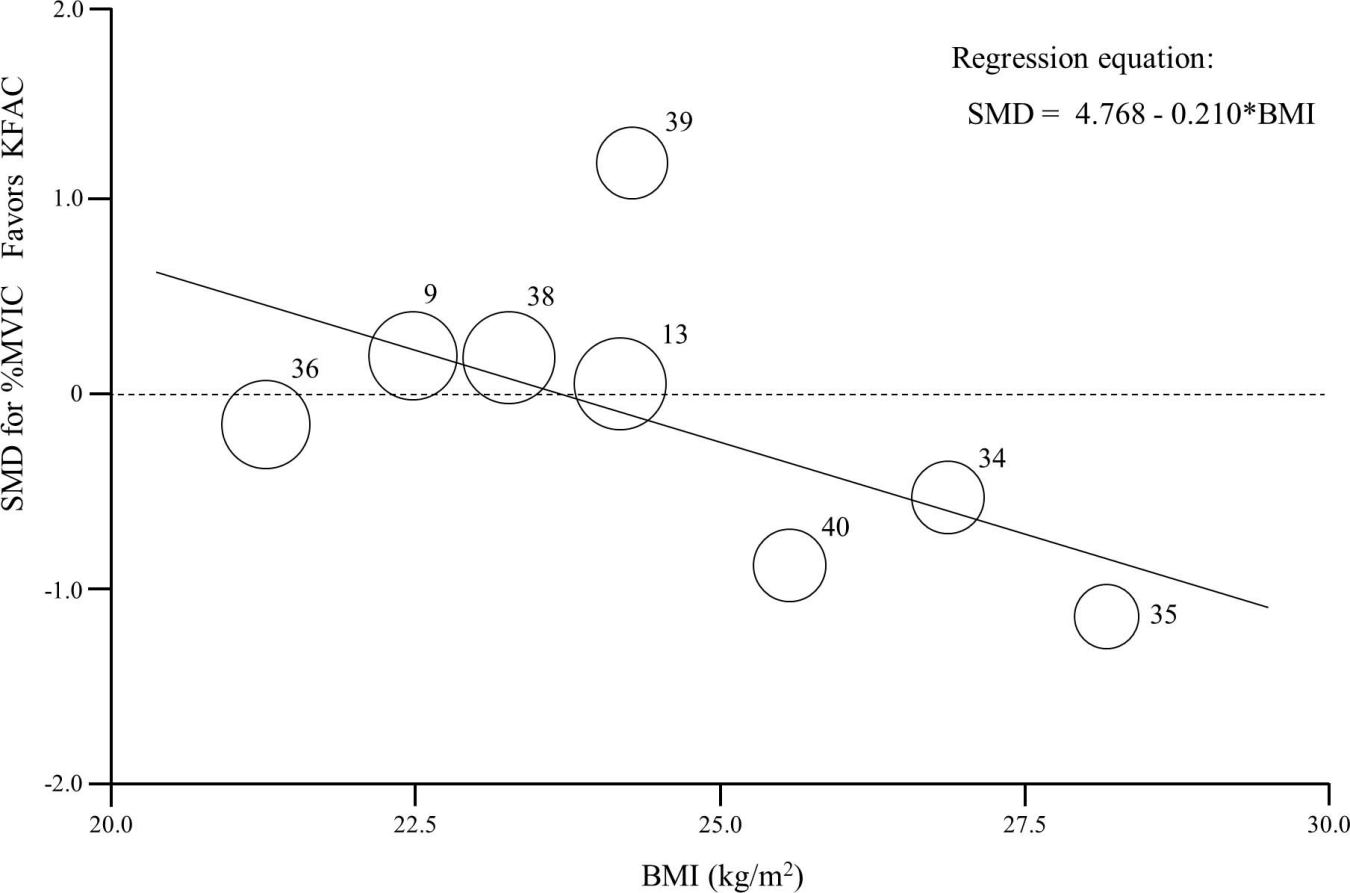
SMD on %MVIC in the included articles, according to BMI, together with a summary of random effects meta-regression analysis. The size of each circle is inversely proportional to weight to correspond to a random effects analysis. The transverse dotted line at 0 represents no difference. Reference numbers are shown. Note that articles by Laufer (2001) and Snyder-Mackler (1989) are not shown because of lack of BMI data. BMI: body mass index; SMD: standardized mean difference; MVIC: maximum voluntary isometric contraction.

**Fig 4:**
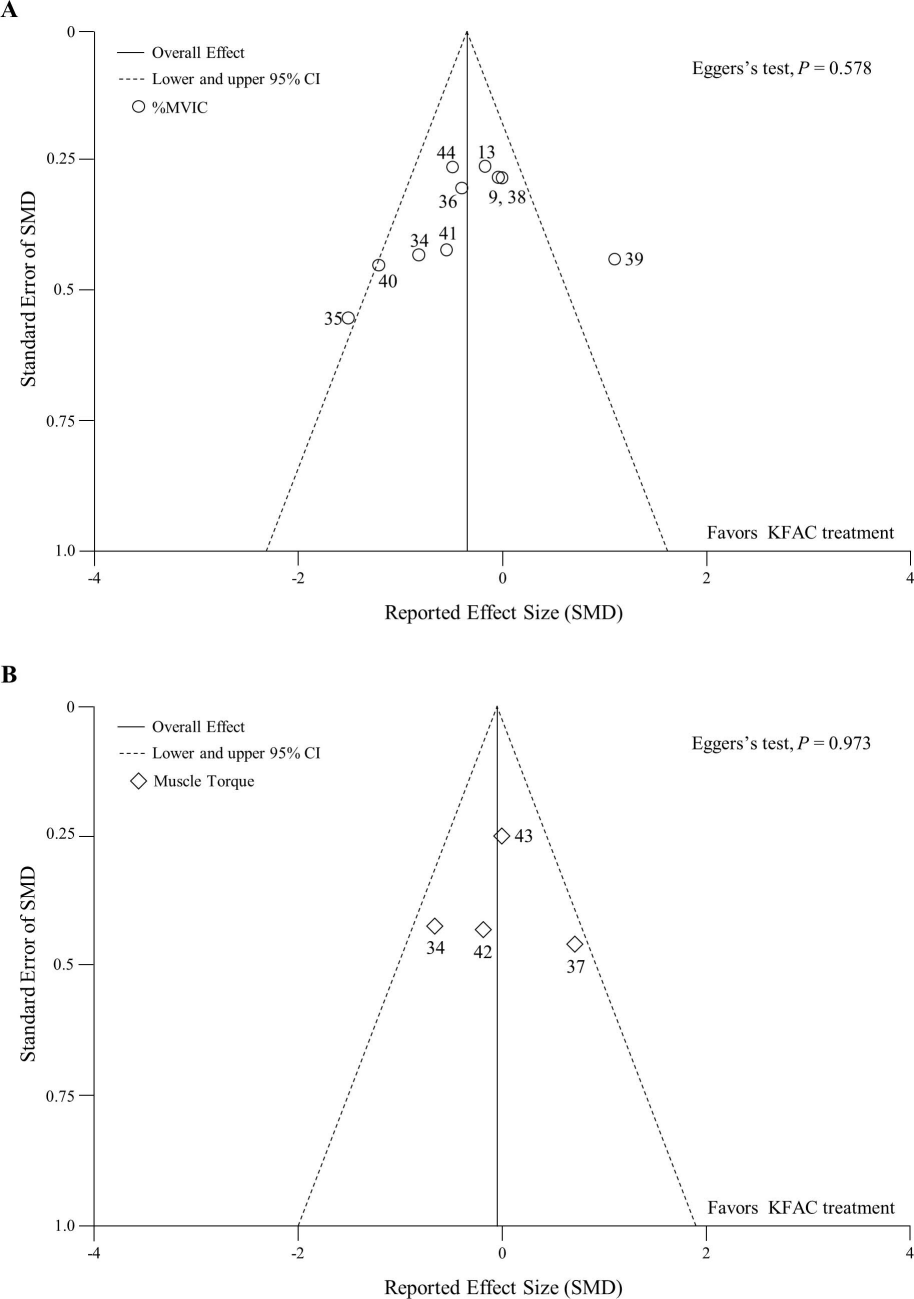
Funnel plot representing publication bias shows a comparison of the effects of PC and KFAC stimulations on %MVIC (A) and muscle torque (B). Egger’s regression test was negative for %MVIC (*P* = 0.578) and muscle torque (*P* = 0.973). Two diagonal lines represent pseudo 95% confidence limits around the summary effect for each standard error on the vertical axis. Reference numbers are shown. CI: confidence interval; KFAC: kilohertz frequency alternating current; SMD: standardized mean difference; MVIC: maximum voluntary isometric contraction.

A random effects meta-regression analysis indicated a potential significant association between SMD for %MVIC and body mass index (BMI) (Table B in S3 File). Fig 3 shows the relationship between SMD on %MVIC and BMI. We examined publication bias by assessing the asymmetry in the funnel plot (Fig 4). Funnel plot asymmetry was not visually observed by the reviewer, and Egger’s regression test was negative for %MVIC (*P* = 0.578) and muscle torque (*P* = 0.973), suggesting an absence of significant publication bias. Numerical data of funnel plots were presented in Table C in S3 File. Non-pooled data of 1 article showed that PC required significantly lower current amplitude (15%) to achieve 10% of MVIC compared to KFAC.[14]

#### 3. 5. 2 Degree of discomfort

**Fig 5:**
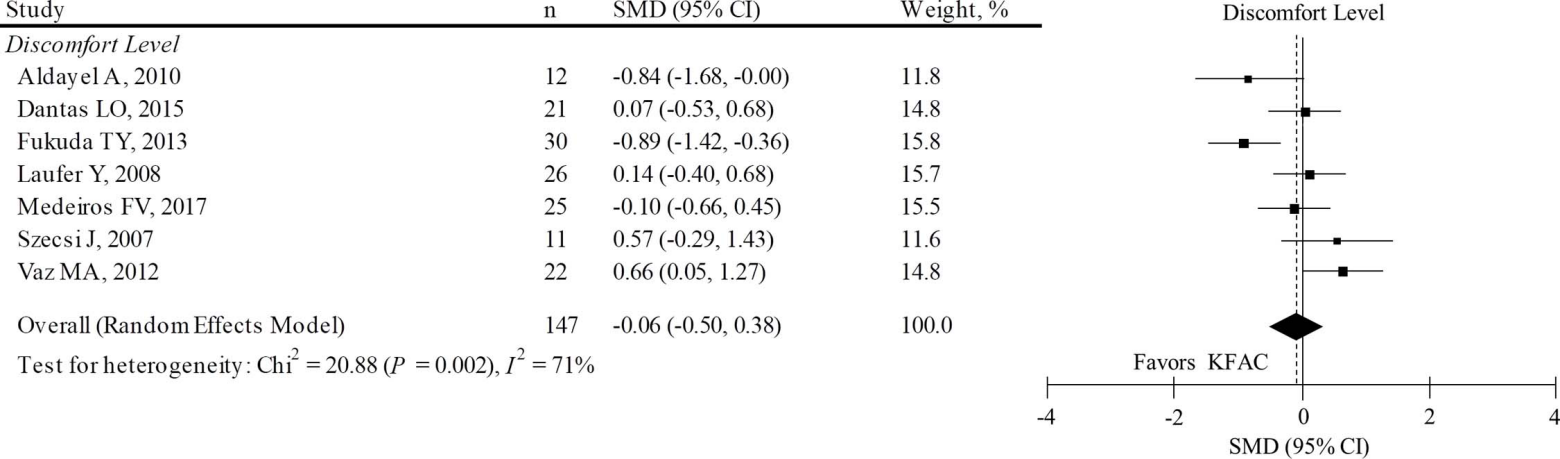
SMD and 95% CI for the discomfort level between PC and KFAC stimulations. The diamond represents the pooled effect size using the DerSimonian-Laird method. The vertical solid line at 0 represents no difference. The vertical dotted line represents pooled SMD. SMD: standardized mean difference; CI: confidence interval; MVIC: maximum voluntary isometric contraction; PC: pulsed current; KFAC: kilohertz frequency alternating current.

**Fig 6:**
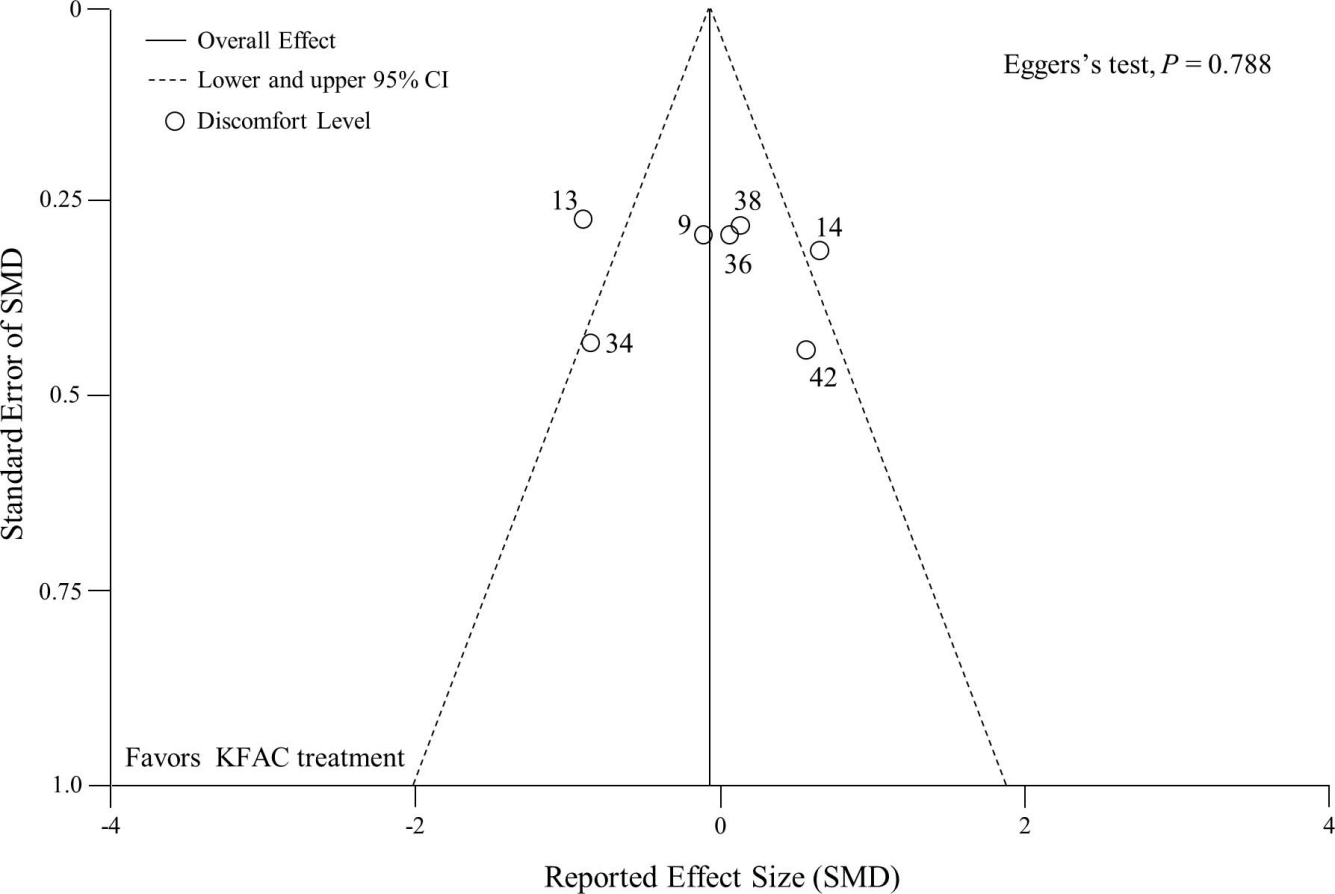
Funnel plot representing publication bias shows a comparison of the effects of PC and KFAC stimulations on discomfort level. Egger’s regression test was negative (*P* = 0.788). Two diagonal lines represent pseudo 95% confidence limits around the summary effect for each standard error on the vertical axis. Reference numbers are shown. CI: confidence interval; KFAC: kilohertz frequency alternating current; SMD: standardized mean difference.

A difference between PC and KFAC on the discomfort level related to electric stimulation (n = 147) was observed in 7 articles.[9, 13, 14, 34, 36, 38, 42] In 1 study,[34] missing data were imputed from available data to estimate pooled SMD for discomfort level. The pooled SMD on the discomfort level was calculated (Fig 5). The results show that KFAC and PC had a comparable effect on discomfort (pooled SMD: -0.06; 95% CI: -0.50, 0.38; *P* = 0.800). Excluding 1 study with imputed data had a minimal effect on muscle torque. Posthoc sensitivity analysis showed comparable results even when 1 article, including subjects with spinal cord injury,[42] was excluded (data not shown). Another posthoc sensitivity analysis revealed that KFAC had significantly lower torque compared to PC when 2 articles, targeting the wrist extensor muscle[38] or hamstrings and gluteus in addition to the quadriceps muscles,[42] were excluded from the meta-analysis (data not shown). However, effects estimates were highly heterogeneous among studies (*I*^*2*^ = 71%; *P* = 0.002). A random effects meta-regression analysis revealed no significant associations between the SMD and any of the characteristics (Table D in S3 File). The publication bias was examined by assessing the asymmetry in the funnel plot (Fig 6). Funnel plot asymmetry was not visually observed by the reviewer, and the Egger’s regression test result was negative (*P* = 0.788), which suggests the absence of significant publication bias. Numerical data of funnel plots were presented in Table E in S3 File. There are two articles[40, 43] that are non-pooled in the meta-analysis. One article reported that the number of subjects who had a negative comment on NMES was higher in KFAC than in PC (66.7% vs. 8.3%).[40] Another article reported that the number of reports of discomfort in each subject was significantly lower in KFAC than in PC (7 vs. 15).[43]

#### 3. 6 Summary of quality of evidence

Table 2 shows a summary of body of evidence according to the GRADE approach. Effects estimates were downgraded in both muscle performance (risk of bias, inconsistency, indirectness, and imprecision) and discomfort level (risk of bias, inconsistency, and indirectness). None of these effects estimates was upgraded. Each meta-analysis scored 1 (very low) on the GRADE approach, which indicates a very little confidence of the effects estimate (i.e., the true effect is likely to be substantially different from the effect estimate).[11]

**Table 2:** Summary of body of evidence according to the GRADE approach: KFAC vs. PC.

Outcome	SMD (95% CI)	Study Design	Number of Subjects	Level of Evidence (GRADE)
*Muscle Performance*				
*%MVIC*	-0.36 (-0.72, 0.00)	× 10 Within-subject	n = 196	⊕ ⊖ ⊖ ⊖ Very low*†‡§
		repeated design		
*Muscle Torque*	-0.04 (-0.53, 0.44)	× 4 Within-subject	n = 65	⊕ ⊖ ⊖ ⊖ Very low*‡§
		repeated design		
*Discomfort Level*	-0.06 (-0.50, 0.38)	× 7 Within-subject	n = 147	⊕ ⊖ ⊖ ⊖ Very low*†‡
		repeated design		

SMD: standardized mean difference; GRADE: Grades of Recommendation, Assessment, Development, and Evaluation.

A negative value of SMD in muscle performance means that PC leads larger muscle torque compared to KFAC. A negative value of SMD in discomfort level means that KFAC is less discomfort compared to PC.

Very low quality: very little confidence that the effects estimate and the true effect are likely to be substantially different from the effects estimate.

*Downgraded for risk of bias (all included studies scored less than 8 points on the Downs and Black scale)

†Downgraded for inconsistency (results were heterogeneous across the included studies: *I*^*2*^ = 66% and 71% on %MVIC and discomfort level, respectively).

‡Downgraded for indirectness (subjects were healthy adults in most of the studies).

§Downgraded for imprecision (clinical action would depend on whether the 95% CI on muscle performance is in the upper or lower boundary)**.**

## 4. Discussion

This systematic review with meta-analysis does not support the hypothesis that KFAC is superior to PC in increasing muscle-evoked torque and lessening discomfort level in adults. However, the quality of evidence was very low according to the GRADE scale because of non-RCT study design, high risk of bias, large heterogeneity, and wide 95% CI of SMD. Thus, no conclusion can be drawn from this meta-analysis. This meta-analysis is the first to compare the effects of KFAC and PC on muscle performance and adverse event in using the GRADE approach, which can shed light on several methodological issues in the included studies. We believe that high quality RCTs that compare the treatment effect between KFAC and PC are needed to facilitate the foundation for effective NMES.

This meta-analysis used comprehensive search strategy including manual research and citation index to identify trials in compliance with recommendation for systematic reviews.[18] Keywords used in the current meta-analysis include those used in the previous meta-analysis,[8] but further keywords were added. These search strategies provide a more comprehensive assessment of relevant articles by adding new findings to the previous review. Indeed, the current meta-analysis added 8 articles[9, 13, 14, 38–40, 42, 43] that were not included in the previous meta-analysis which demonstrated a similar effect of KFAC and PC on quadriceps-evoked torque and discomfort in healthy adults.[8] It should be noted that the mean age of subjects in all the included studies is quite young (26.4 years); thus, our findings may not be applicable to the elderly population, because the elderly have a different force-frequency relationship of quadriceps from healthy adults.[30] However, increasing the number of included trials is variable in the meta-analysis because pooled SMD on muscle performance was statistically more significant than a single trial.[18] It is important to emphasize that our findings differ from the previous meta-analysis that concluded that PC and KFAC have a similar effect on quadriceps muscular function.[8] Indeed, the meta-analysis showed that KFAC leads a lower muscle-evoked torque than PC that is consistent in the sensitivity analysis when only articles targeting the quadriceps muscle in healthy adults were included in the meta-analysis. Since effect estimates would suffer from several bias and confounders as discussed later, the results of the current meta-analysis in favor of PC cannot be explained by sole NMES parameter alone. Generally, longer phase durations generate greater muscle torque at least when monophasic square-wave pulses are used.[45, 46] A recent study also showed that KFAC and PC, with the same phase duration, have similar efficiency for inducing %MVIC, and NMES with longer phase duration induces higher muscle-evoked torque regardless of the carrier frequency,[9] which indicates that phase duration plays an important role on muscle torque. Nevertheless, most of included studies used same phase (pulse) duration, which does not support the theory that KFAC had a short phase duration and produce less muscle torque compared to PC.

In the current meta-analysis for muscle performance, %MVIC and muscle-evoked torque were analyzed separately because these 2 outcomes are different measures. Indeed, pooled SMD is different between 2 outcome measures. While %MVIC is likely to be significantly higher in PC compared to KFAC, the muscle-evoked torque in PC is similar to those in KFAC. However, it should be noted that all of the included studies had a within-subject repeated measure design, indicating that MVIC is the same between subjects in PC and KFAC. Thus, %MVIC and muscle torque may yield similar results. If these 2 outcome measures are integrated into 1 pooled SMD, the overall effect is slightly attenuated but is similar compared to those of %MVIC. All of these data do not support the hypothesis that KFAC is superior to PC in increasing muscle-evoked torque and in lessening discomfort levels in adults.

The risk of bias assessment of Down and Black scale demonstrated that none of the included studies had appropriate blinding or adequate adjustment for confounders. The latter is particularly important because previous experimental studies showed that muscle-evoked torque and discomfort level may be affected by several factors, including, age,[30] sex,[47] skinfold thickness,[48, 49] and NMES parameters,[50, 51] which may attribute to the heterogeneity of effects estimate in the included studies. Although previous meta-analysis[8] also performed quality assessment of included studies using the PEDro scale,[52] this scale would be inappropriate for the assessment of non-RCTs and cannot check whether included studies have adequate adjustment for confounders. To explain inter-trial heterogeneities, we performed random effects meta-regression analysis and found that BMI has a tendency to significantly associate with SMD of MVIC, which indicates its substantial role in causing the heterogeneity of effects estimate. The cause of this relationship is unknown. Although other potential confounders, such as female sex, were not significant factors associated with SMD, meta-regression analysis is well known to often have a low statistical power particular when a meta-analysis has fewer than 10 studies[53]. In the current meta-analysis, the number of articles included for estimating SMD of muscle torque non-normalized MVIC and discomfort level is 4 and 7 articles, respectively. Thus, non-significant result of meta-regression analysis in each variable does not necessarily implicate a lack of impact on effect estimate.[54] Furthermore, it should be noted that only a few articles showed current intensity level in PC and KFAC protocol, which could be a strong confounder[2] even when carrier frequency and pulse duration are the same between two protocols. Given that none of the included studies stratified these potential confounders, investigating the effects of potential confounders on the difference of treatment efficacy between PC and KFAC would be of interest to facilitate a foundation for effective treatment.

A potential factor that was not properly addressed in the included studies is electrode positioning. Muscle impedance is influenced by electrode positioning,[55] and electric stimulation precisely overlies the motor neuron entry point, requiring less current to induce skeletal muscle contraction with possible excitation of the sensory fibers that convey pain.[56] Given the anatomical inter-individual variation of motor neuron location,[57] electric stimulation on subject-specific motor point would evoke muscle torque with less discomfort[58] compared to the electric stimulation on standard motor point described in the traditional textbook.[59] Nevertheless, only one of the 14 included studies considered inter-individual anatomical variability of motor point.[14] Future research should consider inter-individual variability of motor points. This is of particular importance because discomfort levels are high in both PC and KFAC stimulations in the included studies, which could impede the attainment of an optimal muscle contraction.[4]

This meta-analysis provided funnel plots with visual judgment by the reviewer and Egger’s regression test to detect publication bias. The results do not indicate the existence of publication bias, supporting the validity of pooled SMD on muscle performance and discomfort level. However, these results, particularly concerning discomfort level, should be interpreted with caution because when there are relatively few studies, especially less than 10 studies, the sensitivity of the analysis is too low to distinguish chance from real asymmetry.[18]

### 4. 1 Study limitations

First, this meta-analysis included only articles published in English. Indeed, 20 non-English (e.g., Chinese) articles were excluded from the meta-analysis during the full-text screening stage in the current meta-analysis. Thus, language bias may be included in the pooled SMD. It is probable that positive findings are likely to be published in English-language journals, whereas negative findings are published in local journals,[18] the same with German-language literature.[60] Nevertheless, results from funnel plots with Egger’s regression test indicate the absence of publication bias (i.e., funnel plot asymmetry); the finding does not support the theory that only positive findings were published in English journals in the current research question. Furthermore, adding a non-English article,[61] which is included in the previous meta-analysis,[8] had a minimal effect on SMD for discomfort level (data not shown). Second, effects estimate of this meta-analysis was based on non-RCTs that encountered greater bias and several confounders compared to RCTs. Nevertheless, meta-analysis including non-RCTs can provide evidence of effects that are difficult to detect using an RCT, such as adverse events that limit the current dosage and consequently impede the attainment of optimal muscle contraction.[4] Further high quality studies considering potential confounders would be of particular interest. Third, some missing data were imputed from available data such as figure in each article, which may lead to an inaccurate effect estimate. However, the sensitivity analysis showed similar results in muscle performance and discomfort level, indicating that the effect estimates would be robust. Finally, the review processes, such as study selection and data extraction, were performed by a single reviewer, which would yield more errors than the preferred method of independent review by two reviewers.[18] However, to overcome this issue, a single reviewer performed full-text screening twice and citation search of the original record in addition to standard database research.

## 5. Conclusions

This meta-analysis does not support the hypothesis that KFAC is superior to PC in increasing muscle-evoked torque and in lessening discomfort levels in adults. However, no conclusion can be drawn because of a large heterogeneity that cannot be explained by possible confounders and a lack of high quality trials. High quality RCTs comparing the treatment efficacy between PC and KFAC with the consideration of the potential confounders are warranted to facilitate the foundation of an effective treatment.

## Supporting information

S1 File PRISMA checklist. (DOC)

S2 File MOOSE checklist. (DOCX)

S3 File Supplementary methods and tables. (DOCX)

S4 File Certificate of English editing. (PDF)
