# Supplementary material for: Comparison of the effects of kilohertz- and low-frequency electric stimulations: A systematic review with meta-analysis
Source: PLoS One. 2018 Apr 24;13(4):e0195236. doi: 10.1371/journal.pone.0195236 (PMC5915276; doi:10.1371/journal.pone.0195236)
Supplement: S2 File — (DOCX) [file pone.0195236.s002.docx]

A Proposed Reporting Checklist for Authors, Editors, and Reviewers of Meta-analyses of Observational Studies.

| **#** | **Checklist item** | **Reported on page #** |
| --- | --- | --- |
| **Reporting of background should include** | |  |
| 1 | Problem definition | 3–4 |
| 2 | Hypothesis statement | 4 |
| 3 | Description of study outcome(s) | 4 |
| 4 | Type of exposure or intervention used | 3–4 |
| 5 | Type of study designs used | 4 |
| 6 | Study population | 4 |
| **Reporting of search strategy should include** | |  |
| 7 | Qualifications of searchers (eg., librarians and investigators) | 5 |
| 8 | Search strategy, including time period included in the synthesis and keywords | 5 |
| 9 | Effort to include all available studies, including contact with authors | 5–6, eMethod 1 |
| 10 | Databases and registries searched | 5, eMethod 1 |
| 11 | Search software used, name and version, including special features used (eg., explosion) | 5, eMethod 1 |
| 12 | List of citations located and those excluded, including justification | Not applicable |
| 13 | Method of addressing articles published in languages other than English | Not applicable |
| 14 | Method of handling abstracts and unpublished studies | 6 |
| 15 | Description of any contact with authors | Not applicable |
| **Reporting of methods should include** | |  |
| 16 | Description of relevance or appropriateness of studies assembled for assessing the hypothesis to be tested | 5 |
| 17 | Rationale for the selection and coding of data (eg., sound clinical principles or convenience) | 5 |
| 18 | Documentation of how data were classified and coded (eg., multiple raters, blinding, and interrater reliability) | 5 |
| 19 | Assessment of confounding (eg., comparability of cases and controls in studies where appropriate) | 5 |
| 20 | Assessment of study quality, including blinding of quality assessors; stratification or regression on possible predictors of study results | 5–6 |
| **#** | **Checklist item** | **Reported on page #** |
| 21 | Assessment of heterogeneity | 7–8 |
| 22 | Description of statistical methods (eg., complete description of fixed or random effects model, justification of whether the chosen models account for predictors of study results, dose-response models, or cumulative meta-analysis) in sufficient detail to be replicated | 7–8, eMethod 2 |
| 23 | Provision of appropriate tables and graphics | Not applicable |
| **Reporting of results should include** | |  |
| 24 | Graphic summarizing individual study estimates and overall estimate | Figure 2-6 |
| 25 | Table giving descriptive information for each study included | Table 1, |
| 26 | Results of sensitivity testing (eg., subgroup analysis) | 14–17 |
| 27 | Indication of statistical uncertainly of findings | 14–17 |
| **Reporting of discussion should include** | |  |
| 28 | Quantitative assessment of bias (eg., publication bias) | 21–25, Figure 2–6 |
| 29 | Justification for exclusion (eg., exclusion of non-English-language citations) | 25 |
| 30 | Assessment of quality of included studies | 21–25 |
| **Reporting of conclusions should include** | |  |
| 31 | Consideration of alternative explanations for observed results | 26 |
| 32 | Generalization of the conclusions (ie., appropriate for the data presented and within the domain of the literature review) | 26 |
| 33 | Guidelines for future research | 26 |
| 34 | Disclosure of funding source | Not applicable |

From: Stroup DF, Berlin JA, Morton SC, et al. Meta-analysis of observational studies in epidemiology: a proposal for reporting. Meta-analysis Of Observational Studies in Epidemiology (MOOSE) group.

JAMA 2000;283(15):2008–12.
