# Supplementary material for: Comparison of the effects of kilohertz- and low-frequency electric stimulations: A systematic review with meta-analysis
Source: PLoS One. 2018 Apr 24;13(4):e0195236. doi: 10.1371/journal.pone.0195236 (PMC5915276; doi:10.1371/journal.pone.0195236)
Supplement: S3 File — (DOCX) [file pone.0195236.s003.docx]

**Supplementary Online Content**

Method A: Literature Search Keywords 2–4

Method B: Calculation of SMD Using a DerSimonian-Laird Method 5–6

Method C: Detection of Publication Bias Using the Egger’s Test 7

Supplementary References 8

Table A. Downs and Black Scale Score in Each Study 9

Table B. Meta-regression Analysis of Study Characteristics on

the Effects Estimate for the %MVIC 10

Table C. Numerical Data Used in the Funnel Plot for the Muscle Performance 11

Table D. Meta-regression Analysis of Study Characteristics on

the Effects Estimate for the Discomfort Level 12

Table E. Numerical Data Used in the Funnel Plot for the Discomfort Level 13

This supplementary material has been provided by the authors to give readers additional information regarding their work.

**Method A: Literature search keywords**

The following search strategy was employed in each database in accordance with the previous systematic review[^1^](#_ENREF_1). The search in PubMed had a limit of article types depending on the clinical trial.

PubMed

#1 “electric stimulation/adverse effects”[MeSH]

#2 “electric stimulation therapy/adverse effects”[MeSH] AND “Muscle, Skeletal”[MeSH]

#3 “burst modulated alternating current”

#4 “Aussie current”

#5 “Russian current”

#6 “pulsed current”

#7 “neuromuscular electrical stimulation”

#8 “kilohertz-frequency alternating current”

#9 “alternating sinusoidal current”

#10 “middle frequency”

#11 “medium frequency”

#12 “modulated wave”

PEDro

#1 electric stimulation, adverse effects

#2 electric stimulation therapy, adverse effects

#3 burst modulated alternating current

#4 Aussie current

#5 Russian current

#6 pulsed current

#7 neuromuscular electrical stimulation, adverse effects

#8 kilohertz-frequency alternating current

#9 alternating sinusoidal current

#10 middle frequency

#11 medium frequency

#12 modulated wave

CINAHL

#1 “electric stimulation” AND “adverse effects”

#2 “electric stimulation therapy”

#3 “burst modulated alternating current”

#4 “Aussie current”

#5 “Russian current”

#6 “pulsed current”

#7 “neuromuscular electrical stimulation” AND “adverse effects”

#8 “kilohertz-frequency alternating current”

#9 “alternating sinusoidal current”

#10 “middle frequency”

#11 “medium frequency”

#12 “modulated wave”

Cochrane Library (CENTRAL)

#1 “electric stimulation” AND “adverse effects”

#2 “electric stimulation therapy” AND “adverse effects”

#3 “burst modulated alternating current”

#4 “Aussie current”

#5 “Russian current”

#6 “pulsed current”

#7 “neuromuscular electrical stimulation” AND “adverse effects”

#8 “kilohertz-frequency alternating current”

#9 “alternating sinusoidal current”

#10 “middle frequency”

#11 “medium frequency”

#12 “modulated wave”

**Method B: Calculation of pooled SMD using a DerSimonian-Laird method**

We calculated a pooled SMD using a DerSimonian-Laird method according to the previous study[^2^](#_ENREF_2). First, the pooled standard deviation in each study was calculated by using the following formula.

 　　　　　 $N_{i}=n_{1i}+n_{2i}$ (1)

$S_{i}=\sqrt{\frac{(n_{1i}-1)sd_{1i}^{2}+(n_{2i}-1)sd_{2i}^{2}}{N_{i}-2}}$ (2)

*N_i_* = Pooled sample size; n_1_*_i_*: Sample size in the experimental group (post-KFAC treatment); n_2_*_i_*: Sample size in the control group (post-PC treatment); *S_i_*: Pooled standard deviation; sd_1_*_i_*: Standard deviation in the experimental group; sd_2_*_i_*: Standard deviation in the control group.

Subsequently, SMD in each study was calculated by using Hedges’ adjusted g which is similar to Cohen’s d but includes an adjustment for small sample bias.

$SMD_{i}=\frac{m_{1i}-m_{2i}}{S_{i}}\left(1-\frac{3}{4n_{i}-9}\right)$ (3)

$SE\left\{SMD_{i}\right\}=\sqrt{\frac{N_{i}}{n_{1i}n_{2i}}+\frac{SMD_{i}^{2}}{2\left(N_{i}-3.94\right)}}$ (4)

*N_i_* = Pooled sample size; n_1_*_i_*: Sample size in the experimental group (post-KFAC treatment); n_2_*_i_*: Sample size in the control group (post-PC treatment); *m_1i_*: Mean value in the experimental group; *m_2i_*: Mean value in the control group; *S_i_*: Pooled standard deviation; SE (*SMD_i_*): Standard error of *SMD_i_*; *SMD_i_*: Pooled standard mean difference.

Using individual SMD, pooled SMD was calculated using a DerSimonian-Laird method with adjustment for weight in each study.

$SMD_{DL}=\frac{\sum\left(weight_{i}\times SMD_{i}\right)}{\sum weight_{i}}$ (5)

$Weight_{i}=\frac{1}{SE\left\{SMD_{i}\right\}^{2}+\tau^{2}}$ (6)

SE (*SMD_i_*): Standard error of *SMD_i_*; *SMD_i_*: Pooled standard mean difference. *SMD_DL_*: Pooled standard mean difference calculated by a DerSimonian-Laird method.

**Method C: Detection of publication bias using the Egger’s test**

We used a linear regression approach, Egger’s regression[^3^](#_ENREF_3), to measure funnel plot asymmetry. As noted in the previous study[^4^](#_ENREF_4), standard normal deviate (SND) is regressed against the estimate’s precision, the latter being defined as the inverse of the standard error:

$SND=\beta_{0}+\beta_{1}precision$ (7)

This corresponds to a regression analysis of Galbraith’s radial plot[^5^](#_ENREF_5), wherein scatter points lie on the line that runs through the origin at standard normal deviate (intercept *β*_0_ = 0)_,_ with the slope *β*_1_indicating the size and direction effect. The null hypothesis of the test is that *β*_0_ = 0 (no funnel plot asymmetry).

**Supplementary references**

1. da Silva VZ, Durigan JL, Arena R, de Noronha M, Gurney B, Cipriano G, Jr. Current evidence demonstrates similar effects of kilohertz-frequency and low-frequency current on quadriceps evoked torque and discomfort in healthy individuals: a systematic review with meta-analysis. Physiother Theory Pract 2015;31(8):533-9.

2. Deeks JJ, Higgins JP. Statistical algorithms in review manager 5. Statistical Methods Group of The Cochrane Collaboration 2010:1-11.

3. Egger M, Davey Smith G, Schneider M, Minder C. Bias in meta-analysis detected by a simple, graphical test. BMJ 1997;315(7109):629-34.

4. Thompson SG, Higgins JP. How should meta-regression analyses be undertaken and interpreted? Stat Med 2002;21(11):1559-73.

5. Galbraith RF. A note on graphical presentation of estimated odds ratios from several clinical trials. Stat Med 1988;7(8):889-94.

**Table A. Downs and Black scale score of each study**

| Author | Bias | | | | | | |  | Confounding-Selection Bias | | | | | | Total score |
| --- | --- | --- | --- | --- | --- | --- | --- | --- | --- | --- | --- | --- | --- | --- | --- |
|  | *14. Attempted to blind subjects to intervention* | *15. Attempt to blind assessors to intervention* | *16. Clarification for data dredging* | *17. Adjusting for different lengths of follow-up* | *18. Appropriateness of statistical analyses* | *19. Compliance reliability of intervention* | *20. Valid and reliable main outcome measures* |  | *21. Recruitment from the same population* | *22. Recruitment over the same time period* | *23. Patients randomization to intervention group* | *24. Concealed randomization until recruitment complete* | *25. Adequate adjustment for confounders* | *26. Accounted for loss to follow-up* |  |
| ***Within-subject repeated design*** | | |  |  |  |  |  |  |  |  |  |  |  |  |  |
| Aldayel A, 2010 | 0 | 0 | 1 | 1 | 0 | 1 | 1 |  | 0 | 0 | 0 | 0 | 0 | 1 | 5 |
| Aldayel A, 2011 | 0 | 0 | 1 | 1 | 1 | 1 | 1 |  | 0 | 0 | 0 | 0 | 0 | 1 | 6 |
| Dantas LO, 2015 | 0 | 0 | 1 | 1 | 1 | 1 | 1 |  | 0 | 1 | 0 | 0 | 0 | 1 | 7 |
| Fukuda TY, 2013 | 0 | 0 | 1 | 1 | 1 | 1 | 1 |  | 0 | 0 | 0 | 0 | 0 | 1 | 6 |
| Holcomb W, 2000 | 0 | 0 | 1 | 1 | 1 | 1 | 0 |  | 0 | 0 | 0 | 0 | 0 | 1 | 5 |
| Laufer Y, 2001 | 0 | 0 | 1 | 1 | 1 | 1 | 1 |  | 0 | 0 | 0 | 0 | 0 | 1 | 6 |
| Laufer Y, 2008 | 0 | 0 | 1 | 1 | 1 | 1 | 1 |  | 0 | 0 | 0 | 0 | 0 | 1 | 6 |
| Lein DH Jr, 2015 | 0 | 0 | 1 | 1 | 1 | 1 | 1 |  | 0 | 0 | 0 | 0 | 0 | 1 | 6 |
| Medeiros FV, 2017 | 0 | 0 | 1 | 1 | 1 | 1 | 1 |  | 0 | 0 | 0 | 0 | 0 | 1 | 6 |
| Scott W, 2015 | 0 | 0 | 1 | 1 | 1 | 1 | 1 |  | 1 | 0 | 0 | 0 | 0 | 1 | 7 |
| Snyder-Mackler L, 1989 | 0 | 0 | 1 | 1 | 1 | 1 | 1 |  | 0 | 0 | 0 | 0 | 0 | 1 | 6 |
| Szecsi J, 2007 | 0 | 0 | 1 | 1 | 1 | 1 | 1 |  | 0 | 0 | 0 | 0 | 0 | 1 | 6 |
| Vaz MA, 2012 | 0 | 0 | 1 | 1 | 1 | 1 | 1 |  | 0 | 0 | 0 | 0 | 0 | 1 | 6 |
| Ward AR, 2006 | 0 | 0 | 1 | 1 | 1 | 1 | 0 |  | 0 | 0 | 0 | 0 | 0 | 1 | 5 |

All items were scored 1 for fulfilling the criterion or 0 if the criterion was not filled. Publication that did not provide sufficient details to fulfill the criterion were also given a 0 for unable to be determined in accordance of the original index of Downs and Black scale.

**Table B. Meta-regression analysis of study characteristics on the effects estimate for %MVIC**

| Variables | Regression Coefficient (95% CI) | *P*-value |
| --- | --- | --- |
| Age, per year | -0.066 (-0.189, 0.058) | 0.255 |
| Female Sex (0: No, 1: Yes) | 0.002 (-0.011, 0.016) | 0.707 |
| BMI, per unit | -0.210 (-0.501, 0.081) | 0.129 |
| Year of Publication, per year | 0.011 (-0.056, 0.079) | 0.709 |
| Downs and Black Scale Score, per point | -0.138 (-1.173, 0.897) | 0.766 |
| Founding Source (0: No, 1: Yes) | 0.444 (-1.388, 2.278) | 0.583 |

BMI: body mass index; MVIC: maximum voluntary isometric contraction.

**Table C. Numerical data used in the funnel plot for muscle performance**

| Author | SMD | SE | Precision | SND |
| --- | --- | --- | --- | --- |
| *%MVIC* |  |  |  |  |
| Aldayel A, 2010 | -0.810 | 0.429 | 2.333 | -1.890 |
| Aldayel A, 2011 | -1.500 | 0.551 | 1.815 | -2.722 |
| Dantas LO, 2015 | -0.390 | 0.301 | 3.322 | -1.296 |
| Fukuda TY, 2013 | -0.160 | 0.260 | 3.843 | -0.615 |
| Laufer Y, 2001 | -0.480 | 0.260 | 3.843 | -1.845 |
| Laufer Y, 2008 | -0.010 | 0.276 | 3.630 | -0.036 |
| Lein DH Jr, 2015 | 1.110 | 0.444 | 2.253 | 2.501 |
| Medeiros FV, 2017 | 0.000 | 0.281 | 3.564 | 0.000 |
| Scott W, 2015 | -1.220 | 0.449 | 2.227 | -2.717 |
| Snyder-Mackler L, 1989 | -0.540 | 0.418 | 2.390 | -1.291 |
|  |  |  |  |  |
| *Muscle Torque* |  |  |  |  |
| Aldayel A, 2010 | -0.680 | 0.423 | 2.361 | -1.606 |
| Holcomb W, 2000 | 0.720 | 0.464 | 2.154 | 1.551 |
| Szecsi J, 2007 | -0.180 | 0.429 | 2.333 | -0.420 |
| Ward AR, 2006 | 0.010 | 0.250 | 4.000 | 0.040 |

SMD: standardized mean difference; SE: standard error; SND: standard normal deviate.

**Table D. Meta-regression analysis of study characteristics on the effects estimate for discomfort level**

| Variables | Regression Coefficient (95% CI) | *P*-value |
| --- | --- | --- |
| Age, per year | 0.012 (-0.123, 0.147) | 0.831 |
| Female Sex (0: No, 1: Yes) | 0.007 (-0.007, 0.022) | 0.246 |
| BMI, per unit | -0.200 (-0.521, 0.120) | 0.158 |
| Year of Publication, per year | -0.038 (-0.227, 0.151) | 0.628 |
| Downs and Black Scale Score, per point | 0.455 (-0.653, 1.563) | 0.339 |
| Founding Source (0: No, 1: Yes) | 0.407 (-0.965, 1.779) | 0.480 |

BMI: body mass index.

**Table E. Numerical data used in the funnel plot for discomfort level**

| Author | SMD | SE | Precision | SND |
| --- | --- | --- | --- | --- |
| Aldayel A, 2010 | -0.840 | 0.429 | 2.333 | -1.960 |
| Dantas LO, 2015 | 0.070 | 0.291 | 3.439 | 0.241 |
| Fukuda TY, 2013 | -0.890 | 0.270 | 3.698 | -3.291 |
| Laufer Y, 2008 | 0.140 | 0.276 | 3.630 | 0.508 |
| Medeiros FV, 2017 | -0.100 | 0.286 | 3.500 | -0.350 |
| Szecsi J, 2007 | 0.570 | 0.439 | 2.279 | 1.299 |
| Vaz MA, 2012 | 0.660 | 0.311 | 3.213 | 2.121 |

SMD: standardized mean difference; SE: standard error; SND: standard normal deviate.
